# Normothermic Insufflation to Prevent Perioperative Hypothermia and Improve Quality of Recovery in Elective Colectomy Patients: Protocol for a Randomized Controlled Trial

**DOI:** 10.2196/14533

**Published:** 2019-12-20

**Authors:** Edyta Ryczek, Judith White, Ruth Louise Poole, Nicola Laura Reeves, Jared Torkington, Grace Carolan-Rees

**Affiliations:** 1 Cedar Cardiff and Vale University Health Board Cardiff United Kingdom; 2 University Hospital of Wales Cardiff and Vale University Health Board Cardiff United Kingdom

**Keywords:** insufflation, hypothermia, temperature, laparoscopy, humans, peritoneum, carbon dioxide

## Abstract

**Background:**

Perioperative hypothermia during laparoscopy for bowel resection is a risk factor for postoperative medical complications and surgical wound infections. Despite various warming methods used during surgery, a significant number of patients experience perioperative hypothermia. Use of dry, unwarmed insufflation carbon dioxide (CO_2_) during laparoscopic procedures may contribute to this problem. Evidence exists that the HumiGard device, which humidifies and heats CO_2_ for insufflation, can reduce the risk of perioperative hypothermia.

**Objective:**

The aim is to determine if insufflation with warmed, humidified CO_2_ using the HumiGard device, alongside standard perioperative warming techniques, can improve patient recovery, including pain, surgical site infections, complications, and the use of analgesia compared with standard care alone.

**Methods:**

The study is a multicenter, randomized, blinded (patient, surgeon, and assessor), sham device-controlled, parallel group-controlled trial of 232 patients. The study aims to recruit patients undergoing elective laparoscopic, segmental, or total colectomy. Patients will be randomized to receive HumiGard plus standard care or standard care alone (1:1 ratio). The primary outcome is patient-reported quality of recovery, measured by the validated QoR-40 (quality of recovery) questionnaire, from baseline to postoperative day 1. Secondary outcomes include postoperative pain, the incidence of hypothermia, and the rate of postoperative complications.

**Results:**

The information gathered during a small-scale service evaluation at a single hospital was used to inform this study protocol. Before applying for a grant for this full randomized controlled trial, the authors will conduct a feasibility study of 40 patients to ensure that the protocol is feasible and to inform our sample size calculation.

**Conclusions:**

The randomized controlled trial is designed to provide high-quality evidence on the effectiveness of the HumiGard device in potentially reducing the risk of perioperative hypothermia in patients scheduled for laparoscopic colectomy. The results will be used to improve the maintenance of adequate patient body temperature during surgery.

**International Registered Report Identifier (IRRID):**

PRR1-10.2196/14533

## Introduction

### Intraoperative Hypothermia

Patients undergoing colectomy or intra-abdominal surgical procedures are at risk of developing perioperative hypothermia, defined as a core temperature less than 36°C [1]. General anesthesia is one of the contributing factors to the development of hypothermia due to the disruption of normal thermoregulatory responses. Evidence exists that perioperative hypothermia is associated with an increased risk of medical complications, morbid cardiac events, surgical would infections, and extended length of stay in hospital [2].

### Current Standard Practice in the United Kingdom

In the United Kingdom, the National Institute for Health and Care Excellence (NICE) recommends the following: (1) monitoring of patients’ intraoperative temperature every 30 minutes; (2) delaying the induction of anesthesia until the patient’s body temperature is greater than 36°C; (3) warming of intravenous fluids and blood products to 37°C; and (4) for procedures lasting longer than 30 mins, using a forced-air warming device lain on top of patients for warming [3].

Active warming methods do not guarantee that a patient will maintain an adequate body temperature. A recent study by Sun et al [4], which evaluated the core temperature of more than 58,000 actively warmed adults undergoing surgery longer than 60 minutes, showed that nearly half the patients developed hypothermia (body temperature <36°C) during the first hour of the procedure. Based on Lavies et al [5], the use of active warming methods reduced the perioperative incidence of hypothermia, but 53% of patients were still hypothermic in the postoperative phase.

During laparoscopic procedures, standard practice is to use dry, unwarmed CO_2_ to inflate the peritoneum (insufflation). This may contribute to the risk of hypothermia and cause tissue desiccation. Insufflation with unwarmed and dry gas can result in an additional drop in temperature by 1.3°C to 1.7°C [6] and potentially contribute to the risk of perioperative hypothermia.

### Intervention and Study Aims

HumiGard (Fisher & Paykel Healthcare, New Zealand) is a CE-marked medical device that humidifies and heats CO_2_ for insufflation. A meta-analysis of studies that evaluated this type of insufflation demonstrated a significant difference in mean core temperature change, a small beneficial effect on immediate postoperative pain (not at day 1 or 2), with potential impact on the incidence of hypothermia. No difference was observed in patients’ length of stay, analgesic consumption, and procedure duration [7,8]. In February 2017, NICE published guidance on HumiGard for preventing inadvertent perioperative hypothermia [9]. NICE found that the device showed promise, but that more research was needed before a decision could be made on routine adoption within the UK National Health Service (NHS).

This protocol is designed to address the evidence gaps identified by NICE. We aim to determine whether HumiGard used with other standard ways of warming patients results in better outcomes for patients compared with standard care alone. In addition, an economic evaluation will be carried out comparing the cost-effectiveness of HumiGard plus standard care with standard care alone.

## Methods

### Study Design and Population

The study is a multicenter, double-blinded (patient and assessor), sham device-controlled, parallel group randomized controlled trial (RCT). It aims to evaluate if the HumiGard insufflation device, along with standard care, can improve patient-reported quality of recovery (QoR) following laparoscopic colorectal surgery. The study will recruit patients undergoing elective laparoscopic colorectal resection for any pathology. The study is designed to be carried out in the colorectal departments of a minimum of four NHS hospitals across England and Wales.

On receiving the funding to carry out the RCT, the authors will seek a favourable opinion from Health and Care Research Wales and UK Research Ethics Committee. The trial will be registered on ClinicalTrials.gov. The protocol was prepared according to the CONSORT 2010 checklist for reporting parallel group RCTs.

### Eligibility Criteria

The inclusion and exclusion criteria for patients are presented in Textbox 1.

All emergency procedures will be excluded from the study because the presence of sepsis or infection, which affects core temperature, is an additional complication during emergency procedures.

**Patient inclusion and exclusion criteria.**

**Inclusion criteria**

- Adults 18 years or older
- Scheduled for elective laparoscopic, segmental, or total colectomy
- Able to give informed consent

**Exclusion criteria**

- Patients unable to complete study documentation
- Patients that lack the capacity to give informed consent
- Patients with a planned open laparoscopic procedure
- Laparoscopic surgery that is converted to open surgery
- All emergency procedures

### Interventions

During randomization, patients will be allocated to either the treatment arm or control arm of the study at a 1:1 ratio. Patients in the treatment arm will receive humidified and heated CO_2_ insufflation gas into the peritoneal cavity using the HumiGard device. These patients will also receive standard intraoperative warming methods, including warmed fluids and blood products, forced-air warming devices, and warmed blankets at the clinician’s discretion.

Patients in the control arm will be treated with a sham device plus standard intraoperative warming methods, including warmed fluids and blood products, forced-air warming devices, and warmed blankets at the clinician’s discretion. The sham device used in the standard care arm will be the same HumiGard device as is in the intervention arm; however, the sham device will be turned “off” so that the gas delivered to the peritoneal cavity for insufflation is not heated or humidified. The sham device will deliver CO_2_ (as is the case for current standard practice in the hospital) through the HumiGard tubing. The sham device will look and sound the same as the active intervention arm.

### Randomization and Blinding

A member of the research team will telephone the randomization service when a new participant has given signed informed consent to take part in the study to allow randomization to occur. This will occur on day 0 of the study (usually the morning of surgery). Randomization will happen after the patient has consented but before entry into the operating theater. This member of the research team will become unblinded to the allocation of that patient and will not be involved in data collection for that particular patient from the point of randomization onward. Randomization to one of two groups (HumiGard or sham device) will be carried out using a minimization program [10]. Minimization takes into account additional patient information, such as American Society of Anesthesiologists (ASA) grade, gender, and benign or malignant procedure type, to ensure even distribution of patients between treatment groups based on prognostic factors. The unblinded member of the research team will set up the HumiGard device or sham device ready for use according to the allocated group. Neither the patient nor the operating team will be aware of the treatment allocation.

### Clinical Outcomes

The primary outcome measure is the change in patient-reported QoR-40 (quality of recovery) scores from baseline to postoperative day 1 (POD 1).

The QoR-40 is a widely used and validated questionnaire that provides a patient-reported measure of recovery following surgery and anesthesia [11]. The questionnaire includes 40 items separated across five dimensions: patient support, comfort, emotions, physical independence, and pain. The QoR-40 is a reliable and valid tool for assessment of the quality of recovery in patients. It has very high acceptability among patients and is highly sensitive to any clinical changes [12].

The secondary outcomes for this study are:

1. Change in QoR-40 scores from baseline to POD 3.
2. Change in patient-recorded pain scores from baseline to POD 1 and POD 3 using a visual analog scale (VAS). The score will range from 0 to 100.
3. The incidence of hypothermia during the surgery (body temperature drop to 36°C during the procedure as recorded in the patient’s notes).
4. Duration and depth (overall minimum temperature) of hypothermia.
5. The rate of postoperative complications recorded at POD 1, POD 3, at discharge, and at POD 30. The severity will be assessed with the Clavien-Dindo scale, which is widely used for grading the severity of surgical complications in patients [13]. The Comprehensive Complication Index will be used later to create a composite score (0-100) for each patient [14].
6. The incidence of site surgical infections within the first 30 days postsurgery.
7. Length of stay in hospital from procedure to discharge (or until medically fit to discharge).
8. Resource use outcomes, including analgesia (type and dose), use of strategies to maintain perioperative normothermia (eg, warming blanket, fluid warmer), time to discharge, length of procedure, length of recovery time, and readmission to hospital.
9. Cost-effectiveness analysis of the HumiGard device compared with standard care.

### Sample Size Estimation

Assuming a normal distribution of the data, we calculated that 232 patients (116 in each arm) will be required for this study. This number is based on detecting the minimum clinically important difference for the QoR-40 questionnaire of 6.3 with a standard deviation of 14.0 (Myles et al [15]). The standard deviation is high compared with more recent studies, such as Moro et al [16]. The study is powered at the 90% level with a 5% significance level. We allowed for 10% of patients lost to follow-up (for QoR at 24 hours) despite the fact that questionnaires will be issued while a patient will be still in hospital. The final recruitment target of 258 will be split into two cohorts of patients (two arms) of 129 patients each.

### The Study Process and Data Collection

**Figure 1:**
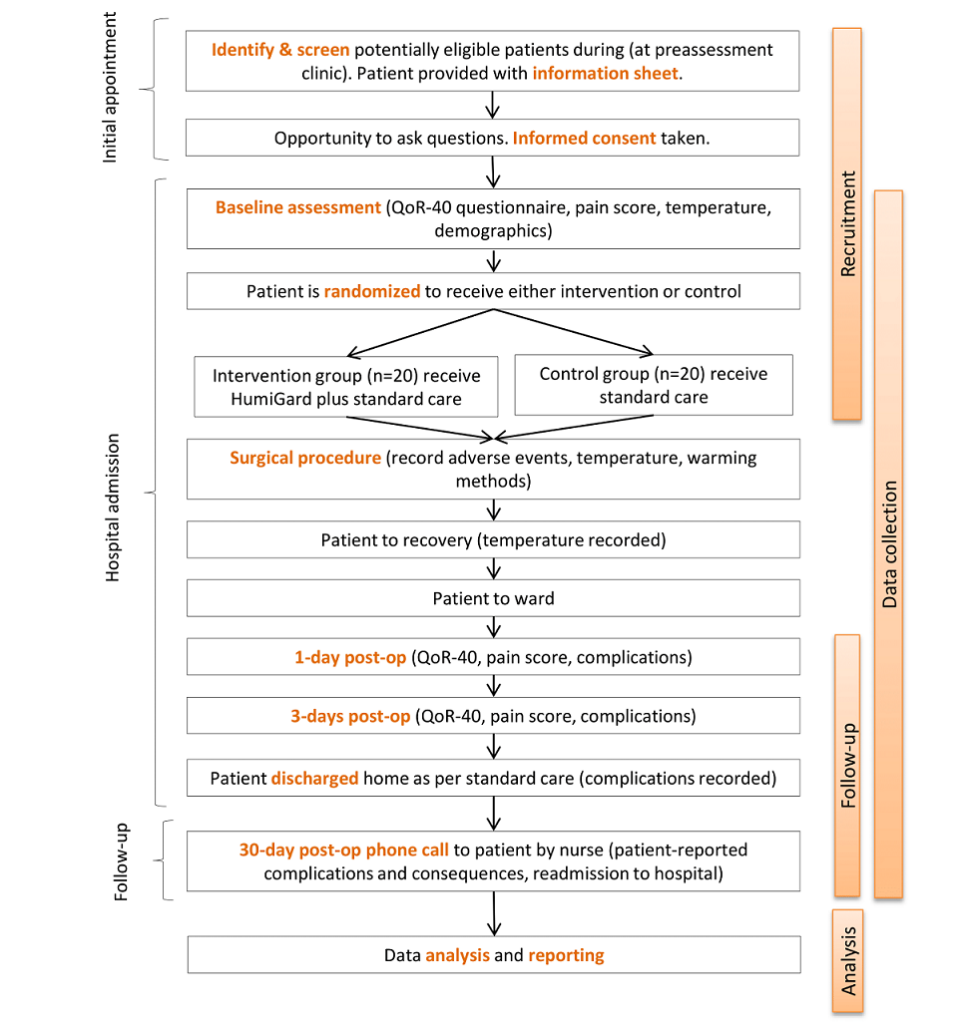
The study process with recruitment, data collection, follow-up, and final analysis.

The flowchart of the study process is presented in Figure 1.

At baseline, patient history, including age, gender, body mass index, smoking status, comorbidities, primary diagnosis, and ASA grade, will be collected. The patient questionnaires and VAS scale will be administered at baseline, POD 1, and POD 3 to patients with no knowledge of their treatment allocation. On arrival at the anesthetic room and during the procedure, the temperature will be measured using a urinary temperature probe.

Patients’ complications (including surgical site infections) will be reported at POD 1, POD 3, upon discharge and at POD 30. All resource use will be routinely recorded at each time point.

### Data Analysis

The statistical analysis plan will be followed for all clinical and economic analyses. Intention-to-treat analysis, the primary analysis method, will take into account minimization factors and sites. Whenever possible, standard errors, confidence intervals, and *P* values will be reported for outcomes.

Assuming a normal distribution of the data, the change in QoR-40 between groups will be analyzed with ANCOVA (analysis of covariance) while controlling for the baseline. The logistic regression for incidence rates will be used to compare the incidence of hypothermia between groups. ANCOVA will be used to compare the duration and depth of hypothermia and the Comprehensive Complication Index scores between groups. The incidence of surgical site infections, length of stay and procedure, and readmission rates will be summarized with descriptive statistics.

### Economic Evaluation

If the intervention is deemed clinically effective, we aim to perform a cost-consequence analysis. The analysis will consider the costs and resource consequences resulting from, or associated with, the use of the HumiGard device plus standard care compared with standard care alone. The model will be produced in MS Excel.

To fully evaluate the impact of the HumiGard device on the current health care system within the United Kingdom, we aim to design the model from an NHS perspective and have a 1-year time horizon. We aim to perform a within-trial analysis with a decision tree that incorporates the rates of complications such as surgical site infections. We will apply the standard discount rate of 3.5%, and the costs of complications and admission rates will be based on NHS reference costs. Differences in staff and bed costs, associated with factors such as length of stay will be included. The cost of analgesia and resource use needed during surgery to maintain normothermia will be incorporated into the cost model.

We will carry out a scenario analysis to validate the model, compare it with other published evidence, and test the impact of changes within the model structure on the base case results. The impact of other published and clinical data will be tested. Deterministic and probabilistic sensitivity analysis will also be performed.

## Results

### Service Evaluation

The preparation of the study protocol was preceded by a small-scale service evaluation carried out in the University Hospital of Wales in Cardiff, United Kingdom. The team investigated the ability and willingness of patients to complete QoR questionnaires before and after laparoscopic colorectal surgery, potential recruitment rates, and informed the design of the data capture tools and database. The study was deemed as nonresearch and approved by Cardiff & Vale University Health Board.

During one month of data collection, seven eligible patients were asked to fill in presurgery (on the day of surgery) and postsurgery (POD 1) questionnaires. The baseline characteristics, intraoperative data, and questionnaire-related completion rates are presented in Table 1. All seven patients filled in the QoR-40 questionnaires at both time points. All patients fully completed the preoperative QoR-40 questionnaire and six of seven patients fully completed all the domains from the postoperative questionnaire (one answer was missing in one of the domains). Six of seven patients completed the preoperative VAS pain question; all patients completed the postoperative VAS pain question.

Statistical analysis of the data collected was not performed due to the low number of patients.

The data obtained from the service evaluation provided “proof of principle” evidence that patients in this setting are amenable to completing the QoR-40.

**Table 1:** Baseline characteristics and intra- and postoperative patient information (N=7).

Characteristic			Participants
**Baseline data**			
	Age (years), mean (range)		50 (19-70)
	**Sex, n (%)**		
		Female	5 (71)
		Male	2 (29)
	**ASA^a^ grade, n (%)**		
		1	1 (14)
		2	4 (57)
		3	2 (29)
	**Type of surgery, n (%)**		
		Laparoscopic elective colectomy	7 (100)
		Open surgery	0 (0)
**Intraoperative data**			
	**Temperature at arrival to theater, n (%)**		
		<36°C	2 (29)
		>36°C	3 (43)
		Unknown	2 (29)
	**Temperature at the end of surgery, n (%)**		
		<36°C	2 (29)
		>36°C	5 (71)
		No change during surgery	2 (29)
		Higher (range 0.3°C-1°C) than at arrival	3 (43)
	Complications (during hospital stay), n		0
	Surgery time (hours), mean (range)		3.33 (1.58-4.17)
**Postsurgery admission**			
	Postanesthesia care unit, n (%)		1 (14)
	Ward, n (%)		6 (86)
	Hospital stay (days), mean (range)		8 (6-15)
**QoR-40^b^ completion rates, n (%)**			
	Fully completed preoperative QoR-40 questionnaires		7 (100)
	Fully completed postoperative QoR-40 questionnaires		6 (86)
**VAS^c^ pain score completion rates**			
	Fully completed preoperative VAS pain score		6 (86)
	Fully completed postoperative VAS pain score		7 (100)

^a^ASA: American Society of Anesthesiologists.

^b^QoR-40: quality of recovery questionnaire.

^c^VAS: visual analog scale.

### Feasibility Study

Following the service evaluation and the preparation of this manuscript, the team received an unrestricted grant for a small feasibility study from Fisher & Paykel Healthcare, the manufacturer of the HumiGard device. The study will use a similar protocol to that described in this publication; however, only 40 patients will be randomized to study arms. The feasibility study will be carried out in the University Hospital of Wales in Cardiff, United Kingdom, and the results will support the funding application for the full-scale RCT described previously.

## Discussion

Systematic reviews [7,8] identified significant weaknesses in the current evidence base for whether using warmed and humidified insufflation gas improves postoperative outcomes for patients. This study is designed to provide information relating to the use of the HumiGard device that is not presently available in the published literature.

Most studies identified did not have the sufficient number of patients required (>100 participants) to show any difference between the comparators. To detect a change in the primary outcome, our sample size will be appropriately powered, and the number of patients will be at least double the size of the cohorts in the published studies. Moreover, the study has a robust, multicenter design with blinding of allocation (patient and outcome assessor).

The control arm will adequately reflect current practice within the United Kingdom and is comparable to the standard practice in other countries. Patients undergoing colorectal surgery have a relatively high risk of developing hypothermia and suffering from postoperative complications due to the length and nature of the procedure as well as existing comorbidities. The inclusion of a control group will help to detect differences between the two arms of the study, if they exist.

More importantly, the study is focused on patient-reported QoR, which will provide an in-depth assessment of patients’ physical recovery, level of emotional stress, and discomfort. It is likely that the QoR-40 will be more sensitive to postsurgery changes in patients’ outcomes than a generic quality-of-life tool because it is focused directly on the time following surgery. Moreover, the “comfort”’ section includes questions regarding shivering and “feeling too cold,” which are relevant to hypothermia. Based on the short service evaluation conducted in University Hospital of Wales in Cardiff, patients are willing to fill in the QoR-40 questionnaires. Thus, QoR-40 is an appropriate tool to use during the proposed RCT.

The clinical trial will provide high-quality evidence necessary to support recommendations about whether HumiGard should be routinely adopted in the hospital setting for patients undergoing surgery. If HumiGard is shown to be effective, patients will benefit from fewer complications and quicker recovery on adoption of this technology. However, if the device is no better than standard care, the health service can avoid unnecessary investments.

One of the major limitations of this or any other medical device study is the short life span of the equipment involved. Medical devices may be expected to provide a long service life; however, it is the manufacturer’s decision when a device is modified or replaced by another model. Unfortunately, the data from clinical trials and other studies are not always transferable, and new evidence must be collected to assess the clinical and cost-effectiveness of devices in which significant modifications have been made.

## Abbreviations

ASA: American Society of Anesthesiologists
NHS: National Health Service
NICE: National Institute for Health and Care Excellence
POD: postoperative day
QoR: quality of recovery
RCT: randomized controlled trial
VAS: visual analog scale
